# Quality and performance measures of strain on intensive care capacity: a protocol for a systematic review

**DOI:** 10.1186/s13643-015-0145-9

**Published:** 2015-11-12

**Authors:** S. Abolfazi Soltani, Armann Ingolfsson, David A. Zygun, Henry T. Stelfox, Lisa Hartling, Robin Featherstone, Dawn Opgenorth, Sean M. Bagshaw

**Affiliations:** Alberta School of Business, University of Alberta, Edmonton, Canada; Division of Critical Care Medicine, Faculty of Medicine and Dentistry, University of Alberta, 2-124 Clinical Sciences Building, 8440—112th Street, Edmonton, Alberta T6G 2B7 Canada; Critical Care Strategic Clinical Network, Alberta Health Services, Edmonton, Canada; Department of Critical Care Medicine, University of Calgary, Calgary, Canada; Alberta Research Centre for Health Evidence (ARCHE), Department of Pediatrics, University of Alberta, Edmonton, Canada

**Keywords:** Quality indicator, Performance measure, Intensive care, Capacity, Health services

## Abstract

**Background:**

The matching of critical care service supply with demand is fundamental for the efficient delivery of advanced life support to patients in urgent need. Mismatch in this supply/demand relationship contributes to “intensive care unit (ICU) capacity strain,” defined as a time-varying disruption in the ability of an ICU to provide well-timed and high-quality intensive care support to any and all patients who are or may become critically ill. ICU capacity strain leads to suboptimal quality of care and may directly contribute to heightened risk of adverse events, premature discharges, unplanned readmissions, and avoidable death. Unrelenting strain on ICU capacity contributes to inefficient health resource utilization and may negatively impact the satisfaction of patients, their families, and frontline providers. It is unknown how to optimally quantify the instantaneous and temporal “stress” an ICU experiences due to capacity strain.

**Methods:**

We will perform a systematic review to identify, appraise, and evaluate quality and performance measures of strain on ICU capacity and their association with relevant patient-centered, ICU-level, and health system-level outcomes. Electronic databases (i.e., MEDLINE, EMBASE, CINAHL, Cochrane Database of Systematic Reviews, Cochrane Central Register of Controlled Trials, Web of Science, and the Agency of Healthcare Research and Quality (AHRQ)—National Quality Measures Clearinghouse (NQMC)) will be searched for original studies of measures of ICU capacity strain. Selected gray literature sources will be searched. Search themes will focus on intensive care, quality, operations management, and capacity. Analysis will be primarily narrative. Each identified measure will be defined, characterized, and evaluated using the criteria proposed by the US Strategic Framework Board for a National Quality Measurement and Reporting System (i.e., importance, scientific acceptability, usability, feasibility).

**Discussion:**

Our systematic review will comprehensively identify, define, and evaluate quality and performance measures of ICU capacity strain. This is a necessary step towards understanding the impact of capacity strain on quality and performance in intensive care and to develop innovative interventions aimed to improve efficiency, avoid waste, and better anticipate impending capacity shortfalls.

**Systematic review registration:**

PROSPERO, CRD42015017931

**Electronic supplementary material:**

The online version of this article (doi:10.1186/s13643-015-0145-9) contains supplementary material, which is available to authorized users.

## Background

The growing and changing demographic profile of the population is projected to contribute to sustained increases in the demand for health services for the foreseeable future. These trends in demographics are accompanied by rising prevalence of persons who have chronic illnesses such as obesity, diabetes mellitus, hypertension, coronary artery disease, and chronic kidney disease [1]. An older population that has greater burdens of chronic disease is more at risk for critical illness [2]. Indeed, patients now receive more complex therapies (e.g., cancer chemotherapy), interventions, or procedures (e.g., complex surgical procedures) for chronic disease. Our ability to effectively treat previously fatal illnesses has advanced and with this so has the demand for critical care services [3].

The matching of critical care service supply with demand is a fundamental requisite for the timely delivery of advanced life support technologies to patients in urgent need. Mismatch in this supply/demand relationship creates *intensive care unit (ICU) capacity strain.* ICU capacity strain is defined as “a temporally varying influence on a given ICU’s ability to provide high-quality care for every patient who is or could become a patient in that ICU on a given day.”[4]. While this definition has face validity, there is uncertainty on how to optimally capture strain on ICU capacity as a quality or performance measure. Indeed, every ICU knows what strain on capacity is when they are experiencing it; however, it has proven challenging to quantify using valid and reliable measures. In preparation for this proposal, we found no robust or validated measures that enable quantification of the immediate or temporal “stress” an ICU experiences due to capacity strain.

What is clear is that ICU capacity strain leads to suboptimal quality of care and directly contributes to higher risk of adverse events, premature discharges, unplanned readmissions, and death [5–11]. When ICU beds are unavailable, care processes may be negatively impacted. The rate of ICU admission refusal increases, and there is greater likelihood of changes to patients’ goals of care towards “do not resuscitate” or “palliation” [12, 13]. This “rationing” is non-transparent, is non-random, and negatively influences quality of care. ICU capacity strain can impede the timely access to critical care services. Patients who have delayed transfer to ICU have significant increases in potentially avoidable morbidity and mortality [12, 14]. A delay in transfer to ICU of ≥6 h from the emergency department has been shown to increase mortality and prolong hospitalization [7]. ICU capacity strain also negatively impacts ICU throughput by increasing the likelihood of premature discharge from ICU [6, 15], increasing the risk of readmission (e.g. “bounce backs”) [11], unnecessary “after-hours” discharges [5, 10, 15], and adverse events [8]. Sustained strain on ICU capacity contributes to the inefficient utilization of health resources and may negatively impact the satisfaction of patients, their families, and frontline providers.

We believe these observations strongly reinforce the importance of identifying valid and robust measures of ICU capacity strain. To address this issue, we propose to perform a systematic review to identify and characterize all available measures of ICU capacity strain. This is the first step in a broader program of research dedicated to the evidence-informed integration of measures of ICU capacity strain into routine practice with the aim to alleviate sustained strain, improve efficiency, and thereby ensure sustainable and consistent high-quality critical care service delivery.

## Objectives

The aim of our review is to systematically evaluate the literature in order to identify proposed measures of ICU capacity strain. The specific objectives are:

1. Create an inventory of quality and performance measures associated with strain on ICU capacity
2. Categorize the measures of ICU capacity strain at the patient level, ICU level, health system level, and human capital level (i.e., human resource related) and across the dimensions of health care quality (i.e., efficient, effective, timely, safe, patient-centered, equity) and the Donabedian framework (i.e., structure, process, outcome).(Tables 1 and 2)

**Table 1:** Sample conceptual framework of measures of ICU capacity strain

Health care quality dimension	Structure	Process	Outcome
Safe	Nurse to patient ratio	ICU-averaged TISS score	Adverse event rate Absenteeism
Effective	ICU census	Delay to ICU admission rate	Adverse event rate Mortality rate
Efficient	ICU discharge protocol	After-hours discharge rate	Unplanned ICU readmission rate
Equity	GOC designation	GOC discussion documentation	Refusal rate Family satisfaction
Patient-centered	Family present at ICU bedside rounds	Time to family conference after admission	HRQL Family satisfaction
Timely	ICU bed occupancy	Queuing Avoidable ICU bed days	Adverse event rate Mortality rate

*ICU* intensive care unit, *TISS* therapeutic intervention scoring system, *GOC* goals of care, intensity, *HRQL* health-related quality of life

**Table 2:** Selected example of potential measures for ICU capacity strain across domains

Domain	Measure
Patient/family level	Avoidable delay to ICU admission
	Mortality rate
	Health-related quality of life among survivors
	Incident disability among survivors
	Family present on ICU rounds
	Time to patient/family conference after admission
	Goals of care discussion/documentation
	Family satisfaction
	Sedation interruption
	Time to extubation after weaning
	Early mobilization
ICU level	ICU census
	ICU occupancy
	Admission ICU census
	Illness severity-adjusted ICU census
	Ventilator-adjusted ICU census
	Queuing
	Avoidable bed days
	After-hours discharge rate/premature discharge rate
	Unplanned ICU readmission rate
	Avoidable adverse events
	Medication error rate
	Duration of inter-disciplinary rounds per patient
	Nurse to patient ratio
	ICU average TISS (workload)
	Respiratory therapist to ventilated patient ratio
Health system/human capital level	Fixed/variable health care costs
	ICU length of stay
	Hospital lengths of stay
	Absenteeism rate
	Personnel turnover rate
	Provider job satisfaction
	Ambulance queue in the ED
	Elective surgery postponement

*ICU* intensive care unit, *TISS* therapeutic intervention scoring system, *ED* emergency department
3. Describe the relevant measurable outcomes associated with ICU capacity strain at the patient-level (i.e., adverse events, mortality, family satisfaction), ICU level (i.e., unplanned readmission, after-hours discharge, avoidable bed days), and health system level (i.e., ICU lengths of stay, hospital length of stay, rehospitalization)
4. Describe the economic and human resource implications associated with ICU capacity strain (i.e., staffing models including surge capacity, absenteeism, burnout, satisfaction)

## Methods

### Study design

A systematic review will be performed to identify and evaluate quality and performance measures of ICU capacity strain using the guidelines from The Cochrane Collaboration and Center for Reviews and Dissemination and described according to the PRISMA-P guideline (available at: http://www.systematicreviewsjournal.com/content/4/1/1) [16] (see Additional File 1).

### Study registration

This systematic review has been registered with PROSPERO (registration # CRD42015017931).

### Criteria for considering studies for this review

#### Inclusion criteria

Studies will be included if they mention *all* of the following broad themes:

1. Intensive or critical care: this theme refers to patients (adults, children and neonates) who are critically ill or at risk for an acute clinical deterioration that may necessitate support in an ICU setting.
2. Quality or performance measure: this theme refers to any measurable variable intended to evaluate the structure, process, or outcome of care provided to patients at risk of being admitted or admitted to ICU.
3. Capacity strain: this theme refers to the evaluation of any measurable variable intended to evaluate the untoward impact at the patient-level, ICU level, health system level, or human capacity level of the stress on ICU capacity.
4. Study design or article source: this theme is intended to include all studies reporting original primary or secondary data encompassing the above mentioned themes, as well as selected gray literature, including administrative reports to governments or health care agencies.

### Exclusion criteria

Studies will be excluded that do not fulfill all of the above criteria, published in a language other than English, and published prior to the year 1990.

### Search methods for identification of studies

The search strategy will be developed in consultation with an expert librarian/information specialist at the Alberta Research Centre for Health Evidence (ARCHE) at the University of Alberta and will undergo subsequent peer review by a second specialized librarian using the Peer Review of Electronic Search Strategies checklist [17]. The information specialist will search electronic databases: Ovid MEDLINE, Ovid EMBASE, CINAHL via EBSCO host, the Cochrane Library including the Cochrane Database of Systematic Reviews, the Cochrane Central Register of Controlled Trials (CENTRAL), and the Web of Science (to also capture operations management literature). Database search results will be from inception to current and restricted to the English language (see Additional File 2).

A combination of the following search themes with the Boolean term AND will be used:

1. intensive care, critical care, critical illness, multi-organ dysfunction, multi-organ failure
2. quality indicator, quality measure, performance measure, quality improvement, quality assurance, quality control, performance improvement, best practice, processes of care, complications, adverse event, medication error, safety, effectiveness, efficiency, appropriateness, outcomes assessment, outcome, audit
3. strain, capacity, occupancy, census, resource, operations management, acuity, rationing, queuing, avoidable, unplanned, readmission, nighttime discharge, absenteeism, burnout, workload, discrete event simulation. Appropriate truncation and wildcards will be used in the search to account for plurals and/or variations in the spelling of search terms. Bibliographic records will be exported to an EndNote X7 (Thomson Reuters, Philadelphia, Pennsylvania) database for screening. Additional sources will be included in the search strategy. The cited and citing references of selected key studies will be searched for relevant articles. Gray literature sources will be searched for clinical practice guidelines, operations management reports, and selected conference proceedings that describe measures of ICU capacity. We will identify and search the websites of relevant organizations (i.e., American Thoracic Society, Society of Critical Care Medicine, Canadian Critical Care Society, European Society of Intensive Care Medicine, Australia and New Zealand Intensive Care Society). We will search the Agency of Healthcare Research and Quality National Quality Measures Clearinghouse (www.qualitymeasures.ahrq.gov) for “ICU capacity”- and “strain”-related quality and performance measures

As part of this process, we will also engage an inter-disciplinary committee of 10–12 stakeholders including knowledge users, decision-makers, and researchers (i.e., policy makers, managers, physicians, nurses, operations management, and quality researchers). This committee will be surveyed for additional quality or performance measures that may be relevant to ICU capacity strain. In addition, this committee will perform a quality assessment of the evidence-base of each identified measure (detailed below).

### Study selection

Potentially eligible articles will be initially identified by having two authors independently review the titles and abstracts of all articles identified by the search. The full text of all articles deemed potentially relevant will be retrieved, and two authors will independently review the full text for inclusion using pre-defined eligibility criteria. Any disagreements that arise will be resolved through discussion or referral to a third party.

### Data extraction

Data will be abstracted from relevant studies using a standardized electronic data collection form. This abstraction will be performed in duplicate by the same two authors. Any disagreements that arise will be resolved through discussion or referral to a third party. The authors of the retrieved studies and/or documents will be contacted for further information as necessary. Methodological quality will be rated using the Newcastle-Ottawa Quality Assessment Scale (NOS) for observational studies and a modified version of BOAS for before-and-after studies [18]. Qualitative studies will be evaluated using the Consensus-based Standards for the selection of health Measurement Instruments (COSMIN) checklist with a four-point scale [5].

Our review proposes three steps to characterize measures of ICU capacity strain: identification, definition, and evaluation.

#### Identification

Measures of capacity strain will be identified through triangulation of all identified articles and related documents or reports and through the survey of inter-disciplinary stakeholder committee.

#### Definition

We will create a summary operational definition and assess its measurement properties (reliability, test-retest), validity (face, construct, criterion)). We will further characterize how each measure is calculated (i.e., simple or complex computation), whether the measure is normalized (i.e., for benchmarking purposes, case-mix differences) and whether the measure is averaged (i.e., capable of continuous assessment of performance over a pre-specified period of observation).

#### Evaluation

Members of the inter-disciplinary stakeholder committee will independently evaluate each measure using the four criteria proposed by the US Strategic Framework Board for a National Quality Measurement and Reporting System according to (i) importance, (ii) scientific soundness, (iii) usability, and (iv) feasibility [19]. Importance will be characterized by whether a measure has evidence of an association with outcomes at the patient, ICU, health system, and/or human capital levels. Scientific soundness will assess how plausible each identified measure’s association with respective outcomes. Usability and feasibility will characterize the logistics and process of implementation of each measure into clinical practice. Each measure will also be evaluated for its potential operational characteristics, for its potential to be integrated into electronic medical records and on its affordability, if applicable.

### Analysis

The primary analysis will be largely narrative. Each measure will be categorized first according to the structure, process, and outcome framework and then by agreed upon domains of evaluation [20, 21]. A comprehensive inventory of measures will be developed and summarized as counts and proportions. These summary counts and proportions will be further stratified based on relevant features such as study design, domains of health care quality, and rank and domains of evidence. When possible, articles and measures will be pooled and further analysis will be performed; however, due to the heterogeneity as well as broad scope of material, it is expected that it will not be possible to pool all measures for analysis. All analyses will be performed using STATA statistical software, version 13 (Stata Corp, College Station, Texas).

## Discussion

Population growth, advances in medical science, and improved capability to support critically ill patients have all translated into a sustained and rising demand for critical care services [3]. This increased demand, however, has not and likely cannot be universally accompanied by an increased supply of critical care resources, which are costly [22]. The costs of expanding critical care extend beyond the “ICU bed” per se and necessitate considerable investment in human resources, specialized equipment (i.e., mechanical ventilators), and supplies to sustainably operate. Moreover, the supply of critical care services is not standardized and is highly inconsistent across jurisdictions for reasons that are not based on evidence-informed scientific assessment of need [23–25]. These observations would imply some degree of redundant inefficiency for the provision of critical care.

Regardless of the reasons, mismatches between demand and supply for critical care services are increasingly encountered [13]. This mismatch on any given day in any given ICU will create a strain on that ICU’s capacity to accommodate the next sickest patient. Accumulating evidence suggests that strained ICUs are at risk of providing suboptimal quality of care [4]. Moreover, such strain drives higher risk of adverse events, premature discharges, unplanned readmissions, and death [7, 8, 10, 12, 15, 26]. Arguably, sustained strain on ICU capacity directly contributes to inefficient utilization of a valuable finite resource. While opening additional ICU beds may seem the simplest response, this is not necessarily sustainable and likely only a short-sighted solution.

In terms of ICU capacity strain, the challenge for health care providers is to clearly understand when and to what extent strain is negatively impacting the quality of care provided and the performance of a given ICU and to readily identify and respond to factors most responsible. There is uncertainty on how to measure ICU capacity strain, and it is unlikely a single measure will satisfy all the potential domains from which strain may originate. Moreover, there may be a limited appreciation for the lateral impact of ICU capacity strain, such as a negative impact on ambulance offloads, emergency department crowding, and postponements of elective surgery. In our preparation for this systematic review, we found little agreement upon measures of capacity strain that would enable quantification of the immediate or temporal “stress” an ICU may be experiencing due to strain on capacity.

Simple, translatable, and easily reportable quality and/or performance measures are needed to diagnose and quantify ICU capacity strain. This is a fundamental initial step towards developing innovative quality improvement interventions aimed to improve efficiency (e.g., access, throughput), avoid waste (e.g., reducing wait time for ICU discharge), and better anticipate impending capacity shortfalls. The next steps in our program focused on ICU capacity strain following completion of this systematic review will involve an evaluation of each identified measure by key stakeholders and experts, followed by prospective appraisal in a clinical context.

### Expected limitations

We anticipate our systematic review will yield a spectrum of heterogeneous measures across a number of potential clinical domains impacted by ICU capacity strain. Similarly, we expect identified measures will be of variable reliability/validity and will have undergone variable implementation and degrees of rigorous assessment. We anticipate there will be limited opportunity for pooled analysis; however, this is not the primary objective of our systematic review. Finally, as aforementioned, we anticipate that a concise “bundle” of measures will likely be necessary to optimally monitor for and characterize strain on ICU capacity, the composition of which will require further prospective evaluation.

## Additional files

Additional File 1:
**PRISMA-P checklist for the systematic review protocol.** (DOCX 17 KB) Additional File 2:
**Proposed example of a peer reviewed search strategy for measures of ICU capacity strain for OVID Medline (version 2, August 11, 2015).** (DOCX 21 KB)

## Abbreviations

ARCHE: Alberta Research Centre for Health Evidence
BOAS: before-and-after studies
CENTRAL: Cochrane Central Register of Controlled Trials
COSMIN: Consensus-based Standards for the selection of health Measurement Instruments
ICU: intensive care unit
NOS: Newcastle-Ottawa Quality Assessment Scale
