# Supplementary material for: Quality and performance measures of strain on intensive care capacity: a protocol for a systematic review
Source: Syst Rev. 2015 Nov 12;4:158. doi: 10.1186/s13643-015-0145-9 (PMC4643503; doi:10.1186/s13643-015-0145-9)
Supplement: Additional File 1: — PRISMA-P checklist for the systematic review protocol. (DOCX 17 KB) [file 13643_2015_145_MOESM1_ESM.docx]

| **PRISMA-P 2015 checklist** | | |
| --- | --- | --- |
| **Section/topic** | **Page #** | **Checklist item** |
| **Title** | | |
| **Identification** | 1 | YES, stated in title. |
| **Update** | 1 | YES, stated in title. |
| **Registration** | 1 | YES, registration number on title page. |
| **Authors** | | |
| **Contact** | 1 | YES, registration number on title page. |
| **Contributions** | 13 | YES, following main body of manuscript. |
| **Amendments** | N/A | Not applicable. |
| **Support** | | |
| **Sources** | 1 | YES, stated on title page. |
| **Sponsor** | 1 | YES, stated on title page. |
| **Role of sponsor/funder** | 1 | YES, stated on title page. |
| **INTRODUCTION** | | |
| **Rationale** | 3-4 | YES, in Background section, pages 3-4. |
| **Objectives** | 5 | YES, in Objectives section, page 5 |
| **METHODS** | | |
| **Eligibility criteria** | 6-7 | YES, stated on pages 6-7. |
| **Information sources** | 7 | YES, stated on page 7 |
| **Search strategy** | 18 | YES, presented in Table 3. |
| **Study records** | | |
| **Data management** | 7 | YES, discussed on page 7. |
| **Selection process** | 8 | YES, discussed on page 8. |
| **Data collection process** | 8 | YES, discussed on page 8. |
| **Data items** | 8-9 | YES, discussed on pages 8-9 and Table 2. |
| **Outcomes and prioritization** | 9 | YES, discussed on page 9. |
| **Risk of bias in individual studies** | 9 | YES, discussed on page 9. |
| **Data** | | |
| **Synthesis** | 9 | YES, discussed on page 9. |
|  | 9 | YES, discussed on page 9. |
|  | 9 | YES, discussed on page 9. |
|  | 9 | YES, discussed on page 9. |
| **Meta-bias(es)** | N/A | NO, not planned. |
| **Confidence in cumulative evidence** | N/A | NO, not planned. |
