# Supplementary material for: Quality and performance measures of strain on intensive care capacity: a protocol for a systematic review
Source: Syst Rev. 2015 Nov 12;4:158. doi: 10.1186/s13643-015-0145-9 (PMC4643503; doi:10.1186/s13643-015-0145-9)
Supplement: Additional File 2: — Proposed example of a peer reviewed search strategy for measures of ICU capacity strain for OVID Medline (version 2, August 11, 2015). (DOCX 21 KB) [file 13643_2015_145_MOESM2_ESM.docx]

**Additional File 2:** Proposed example of a peer reviewed search strategy for measures of ICU capacity strain for OVID Medline (version 2, August 11, 2015)

1. exp Critical Care/

2. exp Critical Care Nursing/

3. Critical Illness/

4. Intensive Care Units/

5. exp Intensive Care Units, Pediatric/

6. Multiple Organ Failure/

7. critical [care.tw](http://care.tw/),kf.

8. (critical* adj2 (department* or unit*)).tw,kf.

9. critical* ill*.tw,kf.

10. intensive care*.tw,kf.

11. (intensive adj2 (department* or unit*)).tw,kf.

12. intensivist*.tw,kf.

13. (ICU* or NICU* or PICU*).tw,kf.

14. (multi* organ adj (disfunction* or dis function* or dysfunction* or dys function* or failure*)).tw,kf.

15. (multi* system adj (disfunction* or dis function* or dysfunction* or dys function* or failure*)).tw,kf.

16. or/1-15 [Combined MeSH terms and keywords for critical care]

17. Delivery of Health Care/

18. Efficiency/

19. Efficiency, Organizational/

20. Health Services Research/

21. exp Medical Audit/ and (ec or og or sn or ut).fs.

22. "Outcome Assessment (Health Care)"/

23. patient outcome assessment/

24. exp Program Evaluation/

25. Quality Assurance, Health Care/

26. Quality Improvement/

27. Quality Indicators, Health Care/

28. Quality of Health Care/

29. (bench mark* or benchmark*).tw,kf.

30. (delivery adj2 health*).tw,kf.

31. (efficien* adj2 (assess* or assurance* or evaluat* or improv* or indicat* or measur* or test*)).tw,kf.

32. (health* adj2 service* research).tw,kf.

33. (performance adj2 (assess* or assurance* or evaluat* or improv* or indicat* or measur* or test*)).tw,kf.

34. (outcome* adj2 (assess* or assurance* or evaluat* or improv* or indicat* or measur* or test*)).tw,kf.

35. (program* adj2 (assess* or assurance* or evaluat* or improv* or indicat* or measur* or test*)).tw,kf.

36. or/17-35 [Combined MeSH terms and keywords for quality]

37. Absenteeism/

38. Bed Occupancy/

39. Burnout, Professional/

40. exp Hospital Bed Capacity/

41. exp Health Services Accessibility/

42. exp Job Satisfaction/

43. exp Patient Discharge/

44. exp Patient Readmission/

45. exp Resource Allocation/

46. Waiting Lists/

47. absenteeism*.tw,kf.

48. (access* adj2 service*).tw,kf.

49. ((admiss* or admit*) adj2 (deni* or deny or refus*)).tw,kf.

50. ((allocat* or distribut*) adj2 resource*).tw,kf.

51. ((bed or beds) adj3 (capacit* or occupan*)).tw,kf.

52. (burn* out or burnout).tw,kf.

53. (capacity adj2 (limit* or strain*)).tw,kf.

54. ((job or jobs or profession*) adj2 satisf*).tw,kf.

55. ((pre mature* or premature*) adj discharg*).tw,kf.

56. queu*.tw,kf.

57. rationing*.tw,kf.

58. (re admission* or readmission*).tw,kf.

59. wait* list*.tw,kf.

60. or/37-59 [Combined MeSH terms and keywords for capacity strain]

61. and/16,36,60 [Combination of ICU, quality and capacity terms]

62. exp animals/ not humans.sh.

63. 61 not 62 [Human filter] (1864)

64. limit 63 to yr="1990-current" [Date range limit]

65. limit 64 to english [language limit]

66. remove duplicates from 65
